# An Ethnobotanical Study in Kırşehir (Türkiye)

**DOI:** 10.3390/plants13202895

**Published:** 2024-10-16

**Authors:** Gizem Emre, İsmail Şenkardeş, Kaan İşcan, Oltan Evcimen, İlknur Yılmaz, Osman Tugay

**Affiliations:** 1 Department of Pharmaceutical Botany, Faculty of Pharmacy, University of Marmara, 34854 İstanbul, Türkiye; isenkardes@marmara.edu.tr; 2 Department of Animal Breeding and Husbandary, Faculty of Veterinary Medicine, University of Erciyes, 38280 Kayseri, Türkiye; kaan@erciyes.edu.tr; 3 Department of Sociology, Faculty of Letters, University of Erciyes, 38280 Kayseri, Türkiye; oltanevcimen@erciyes.edu.tr (O.E.); ilknurylmz93@gmail.com (İ.Y.); 4 Department of Pharmaceutical Botany, Faculty of Pharmacy, University of Selcuk, 42250 Konya, Türkiye; otugay@selcuk.edu.tr

**Keywords:** ethnobotany, folk medicinal plants, wild edible plants, Kırşehir, Türkiye

## Abstract

A comprehensive ethnobotanical study was carried out in the province of Kırsehir, in the Central Anatolia region. The result of that study is this publication containing information about the main traditional folk medicine, wild food plants, and other plant uses in the area. Our goal was to collect, identify, and provide information about plants traditionally used by the local population. This inquiry focused on the plant specimens obtained during field work. Data were gathered through open and semi-structured interviews conducted with local individuals, and use report (UR) values were computed. A total of 79 taxa of plants used in folk medicine, belonging to 33 families, were identified in this study. Of these, 67 taxa were wild and 12 were cultivated. The most common families were Asteraceae, Lamiaceae, and Rosaceae. Based on the results of this study, 77 taxa with ethnobotanical uses were recorded. The plants were used as folk medicine (45 taxa), as food (46 taxa), and for other purposes (34 taxa). This study determined that plants are still traditionally used in the region.

## 1. Introduction

Local communities possess traditional ecological knowledge (TEK) which offers valuable insights into the cultural and ecological significance of many plant species, as well as their potential for sustainable utilization [1,2]. One of the important components of traditional ecological knowledge is ethnobotany [3]. The documentation of ethnobotanical knowledge is highly valuable from a scientific perspective, especially in the current era characterized by rapid social change, declining plant biodiversity, and loss of traditional knowledge regarding wild plant usages [4,5].

In recent decades, there has been a significant increase in global initiatives aimed at safeguarding ethnobotanical knowledge, particularly in Europe, Mediterranean countries, and Türkiye [5,6,7,8]. However, it seems that these initiatives are mostly related to medicinal and wild food plants. In Türkiye, ethnobotanical studies are carried out with the efforts of researchers and the support of the government. This study has been supported by The Turkish Ministry of Agriculture and Forestry [7]. Kırşehir Province has seen only two ethnobotanical studies [9,10], both conducted in the same district. With its rich cultural and biological heritage, Kırşehir demands further investigation from an ethnobotanical perspective. The aim of this study is to carry out a comprehensive ethnobotanical study in the region and examine how the role of plants in human life has been determined, and how traditional knowledge can be used as a resource to help future generations.

## 2. Results and Discussion

**Figure 1:**
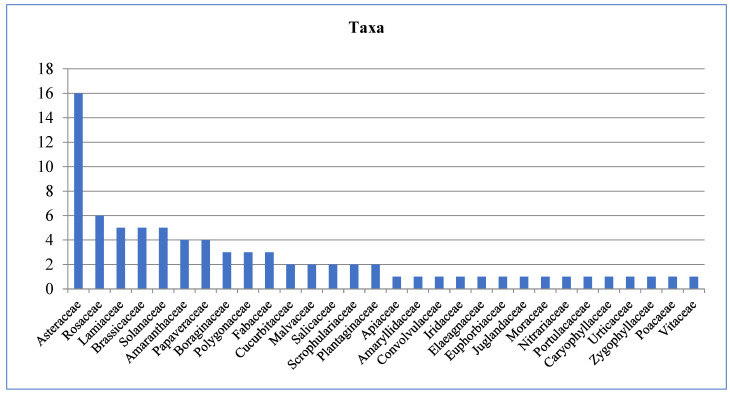
Number of taxa per family.

We have created a comprehensive list of 79 taxa belonging to 33 distinct families. Of these taxa, 67 were wild and 12 were cultivated. The family Asteraceae had the highest representation, with a total of 16 plant species. This was followed by Rosaceae and Lamiaceae, with six and five species, respectively (Figure 1).

As a conclusion of the study, 45 plants were recorded to be used in medicinal treatment, while 46 plants were consumed as edible and 34 plants were classified in other purposes. Also, the data collected in the region has been compared with the neighboring provinces [9,10,11,12,13,14,15,16,17,18,19,20,21,22,23,24,25] (Table 1).

A total of 1291 use reports were identified in terms of ethnobotanical uses of plants in the study area. According to use reports, the most popular plants were *Peganum harmala* L. (89 URs), *Malva neglecta* Wallr. (78 URs), *Vitis vinifera* L. (62 URs), and *Malva sylvestris* L. (55 URs). It has been determined that *P. harmala* is used in medicinal and other plant usages; *Malva neglecta-M.sylvestris* are used in medicinal and edible usages; and the *V. vinifera* is used in medicinal, edible, and other plant usages.

During our studies it has been observed that the medical and nutrient plants are still used commonly in the region. However, today it has been determined that their usage as dyes and toys have decreased compared to other usage categories. It has been determined that animal origin usage has been rarely used today.

From a total of 99 people interviewed in the settlements during the field studies, 63 were aged 50–70. It was determined that the people aged 50–70, from whom the most information was obtained, were mostly primary school graduates.

*Astragalus melanophrurius* Boiss., *Centaurea urvillei* DC. subsp. *stepposa* Wagenitz, *Convolvulus galaticus* Rostan ex Choisy, and *Papaver pilosum* Sibth. et Sm. are endemic species (Table 1). The distribution of these endemic species is throughout Central Anatolia. These endemic plants used ethnobotanically in the study area are not in the CR, EN, or VU categories, and their risk of extinction is almost non-existent [11].

### 2.1. Medicinal Plants

The plants used for medicinal purposes in Kırşehir are presented in Table 1 and arranged alphabetically according to their botanical names, together with any further relevant information. During this study, 59 plant specimens were collected in the research area. Of these, 45 medicinal plant species belonging to 22 families were identified, of which 36 species were wild and 9 species were cultivated. The most common medicinal plant families were Asteraceae (45.5%), Rosaceae (27.3%), Solanaceae (22.7%), and Lamiaceae (18.2%).

Based on a total of 419 use reports, the plant parts used to treat ailments were leaves (27.6%), aerial parts (23.8%), subterranean parts (9.5%), fruit (8.3%), and other parts such as latex, resin, and the bark of a stem (30.8%).

A total of 112 medicinal uses were recorded. The preparation methods were infusion (18.8%), direct application without any preparation (18.8%), application after crushing (17.8%), decoction (14.8%), and other common methods (39.8%).

Remedies were mainly administered externally (55.4%) (Table 1).

Remedies were sometimes prepared with additional components: eggs, molasses, melted beef suet, flour, henna, or bulgur.

Of the medicinal plants used for veterinary purposes, *Cydonia oblonga* Mill. and *Elaeagnus angustifolia* L. were used to treat both humans and animals. While leaves of *C. oblonga* are used internally against diarrhea, the leaves of the *E. angustifolia* are used externally against abdominal pain in animals.

When our own study was compared with two previous studies [9,10] conducted in the same city, the use of ten plants in treatment was determined. These planst are: *Achillea biebersteinii* Afan., *Cichorium intybus* L., *C. oblonga*, *Gundelia tournefortii* L., *Malva neglecta*., *M. sylvestris*, *Peganum harmala*, *Teucrium polium* L., *Tribulus terrestris* L., and *Vitis vinifera*. It has been determined that the uses of *C. intybus*, *M. neglecta*, *T. polium*, *T. terrestris*, and *V. vinifera* in the treatment of these plants are the same.

**Figure 2:**
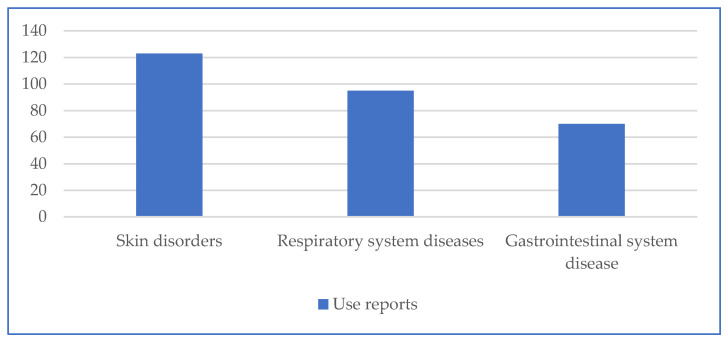
Number of use reports for each ailments category.

According to our results, *Malva neglecta* (51 URs), *T. polium* (29 URs), *R. canina* (28 URs), *Euphorbia macroclada* Boiss. (27 URs), and *C. oblonga* (25 URs) were the most cited plant species for medicinal uses. The highest number of use reports (123) were for skin disorders, followed by respiratory (95) and gastrointestinal (70) ailments (Figure 2).

To the best of our knowledge, *Malva neglecta* and *Rosa canina* L. were found to be the most commonly used herbal medicinal plants and were recorded at ten localities in Kırşehir and its environs in accordance with previous studies [9,10,12,13,14,15,16,17,18,19,20,21,22,23,24,25], as shown in Table 1. *M. neglecta* is used in the treatment of wounds, hemorrhoids, gynecological diseases, and abdominal pain, and *R. canina* is used for cold, cough, and diabetes in our research area and the Central Anatolia region.

Analysis of the most often utilized plants in the area showed that similar plants are employed for the same purposes in neighboring provinces and European countries [9,10,12,13,14,15,16,17,18,19,20,21,22,23,24,25,26,27,28,29,30,31,32,33,34,35,36,37,38,39,40,41,42,43,44,45].

A poultice of *M. neglecta* and bulgur is used for abdominal pain. A poultice of *M. neglecta* is also used to treat wounds. These two usages are similarly employed in surrounding places [17,20]. *M. neglecta* is also used in the Balkans and Europe to treat coughs and bronchitis [26]. Similarly, *Malva sylvestris* is commonly utilized in medical treatment [3,4,5,6,7,8,9,10,11,12,13,14,15,16,17,18,19,20,21,22,23,24,25,26,33,34].

A poultice of the leaves of *M. sylvestris* is used to treat wounds. The leaves of *M. sylvestris* have been documented to possess strong anti-inflammatory, antioxidant, anti-complementary, anti-carcinogenic, and skin tissue integrity properties. Furthermore, a recent study has confirmed the anti-ulcerogenic property of the aqueous extract. It was found to be more efficacious than cimetidine, a strong medication prescribed to treat gastric ulcers [34].

*Teucrium polium* is widely utilized in the region, particularly as an appetite stimulant, and its use has been documented in nearby towns [9,12,15,20]. *T. polium*, a wild flowering perennial widely distributed in North Africa, Europe, and South-Western Asia, is used to treat various disorders. In Iran, it is used for inflammation, gastrointestinal disorders, rheumatism, and diabetes [35]. In Jordan, it is used for diabetes, rheumatism, kidney stones, and fever [36]. In Mediterranean countries, it is used for several disorders. It is used for gastrointestinal disorders in Bosnia and Herzegovina [35,36,37,38,39] and as an anti-icteric, anthelmintic, and tonic in Spain [39]. *T. polium* contains various classes of compounds, i.e., monoterpenes, diterpenes, fatty acid esters, sesquiterpenes, polyphenolics, and flavonoids. It has been reported that these compounds have anti-proliferative, anti-diabetic, anticarcinogenic, and pro-apoptotic properties [40].

*Rosa canina* fruit infusion is widely utilized in the region and other provinces to treat colds [13,14,17,19,23,24]. Reports indicate that it has been used to treat colds in other countries [5,6,28,29,30,42], as *R. canina* has antioxidant and anti-inflammatory properties [39].

*Cydonia oblonga* is commonly ingested as a decoction to help relieve shortness of breath and cold. These applications are similar to therapeutic applications in neighboring areas and other countries [5,6,14,19,24,42,43]. There is scientific evidence that quince possesses antimicrobial properties that specifically target gram-positive bacterial infections [44].

The latex of the *Euphorbia* plant is often used, locally and in the surrounding areas [15,23], to treat skin diseases. Its biological properties and pharmacological functions include antibacterial, antioxidant, free radical scavenger, cytotoxic, tumor, anti-inflammatory, healing, hemostatic, anti-angiogenic, insecticidal, genotoxic, and mutagenic [45].

### 2.2. Edible Plants

We documented 566 use reports detailing the application of 45 taxa for culinary purposes (Table 1).

The plant taxa most frequently indicated for food were *Vitis vinifera* (56 URs), *Polygonum cognatum* Meissn. (38 URs), *Malva sylvestris* (32 URs), and *M. neglecta* (28 URs).

**Figure 3:**
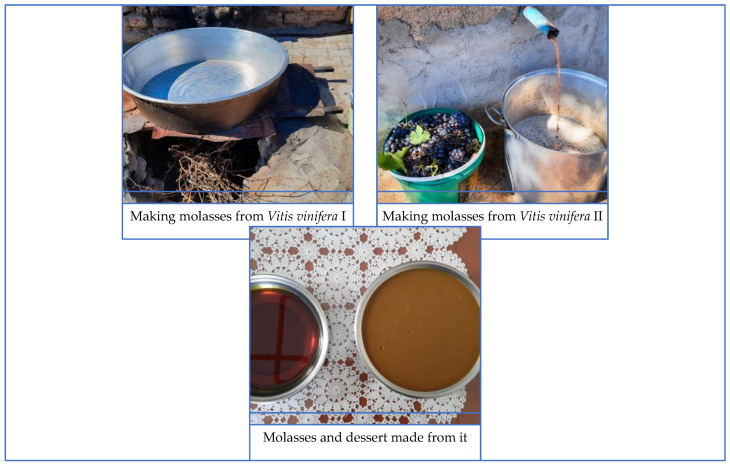
Dessert and molasses made from *Vitis vinifera* (İ. Yılmaz, 2021).

*Vitis vinifera* is widely cultivated in numerous locales in the area, and molasses is produced from its fruit. This molasses is frequently utilized in the preparation of sweets (Figure 3).

**Figure 4:**
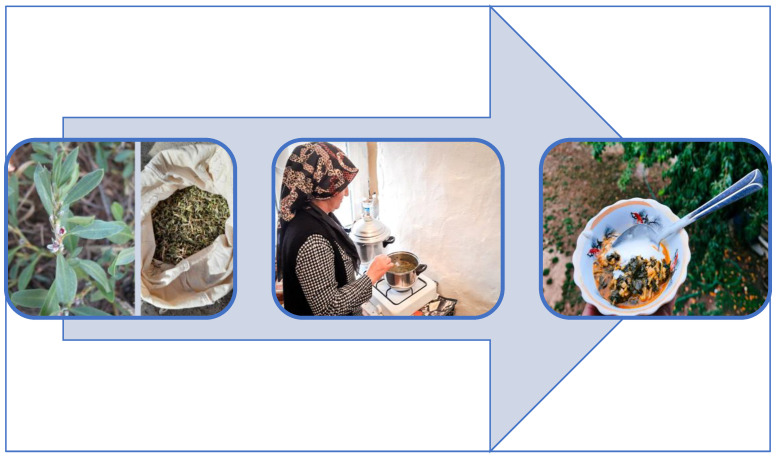
*Polygonum cognatum* and a dish made from this plant (İ. Yılmaz, 2021).

*Polygonum cognatum*, an extensively distributed species, is a popular wild plant known for its nutritional value. Once food is prepared, inhabitants gather and dry it throughout the spring and then consume it during the winter. Several participants go so far as to collect the plant when it is still fresh and store it in a deep freezer (Figure 4). It has been determined that this plant, prevalent in the inland regions of Türkiye, is consumed in similar ways in the neighboring areas [9,10,13,16,18,20,22].

*Malva neglecta* and *M. sylvestris*, frequently utilized in local remedies, were discovered to be consumed cooked, raw as dolma, and as snacks (for children). Both plants have been identified as wild food plants in surrounding regions and in Europe [5,9,12,16,18,20,22,44,45,46,47,48,49,50].

Plants that lack therapeutic properties but are commonly ingested as infusions, such as recreational tea, are also taken into account in this context (Table 1).

It was learned from interviewees that the endemic *P. cognatum, Centaurea urvillei* DC., and *Papaver pilosum* Sibth. et Sm. plants are used as wild food sources (Table 1).

It was determined that the majority of the plants used in this region are both raw and cooked. It was ascertained that they are ingested in the form of spices, baked products (borek), coffee, and jam.

### 2.3. Other Plant Usages

Upon examining the various uses of plants indicated by the informants, it becomes apparent that some roles are prominent: dyes, brooms, amulets, musical instruments, sticks, fodder, and fuel. There were a total of 248 URs (Table 1).

**Figure 5:**
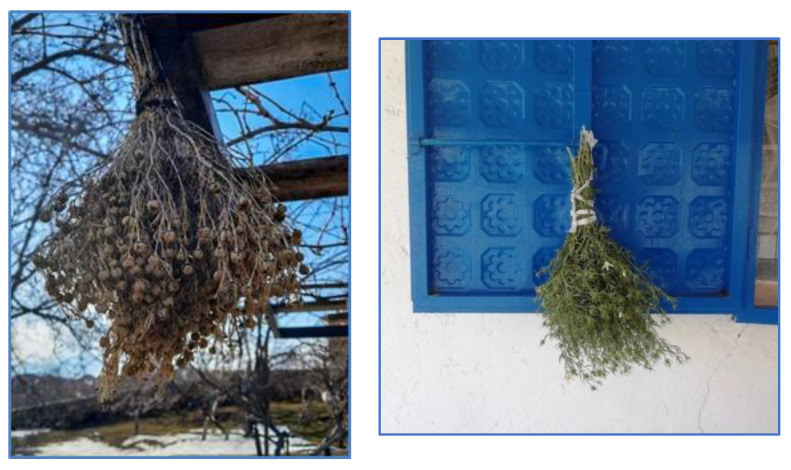
*Peganum harmala* used as amulet (İ. Yılmaz, 2021, O. Tugay, 2022).

One of the main applications of *Peganum harmala* (66 URs), which is abundant in the area, is as an amulet. Local residents place this plant both inside and outside of their houses, in their barns and in their gardens or fields when planting new crops. The use of this element, which is the Irano-Turanian element, as an amulet or as incense is also recorded elsewhere [10,12,51,52] (Figure 5).

A crucial aspect is that the plant is situated in a conspicuous location. In addition, local people believe that this plant will help them find solutions to their problems with this plant. This belief is expressed in a poem:

“Üzerlik yüzbin ellik
Başında yeşil terlik
Göz edenlerin gözü çıksın
Atmış, yetmiş dağlara çıkmış gitmiş
Üzerliksin, üzerliksin cefasın
Her dertlere devasın
Karga gelsin, kavursun
Bütün dertlerimiz savrulsun”

“Harmal seed, a hundred thousand hands
Green cap is on his head.
The one with the evil eye, may his eye fall out
Sixty, seventy went to the climb the mountain
You are the harmal seed, you are the harmal seed, you are the pain
You heal all the troubles
Let the crow come, let the crow blight all the troubles
And all the troubles are blown away”

*Elaeagnus angustifolia* is another plant commonly used as an amulet (18 URs). Charms crafted from the branches of this plant are utilized in the region to ward off the evil eye.

The leaves and outer covering of the *Juglans regia* L. (40 URs) plant are utilized as a coloring agent.

**Figure 6:**
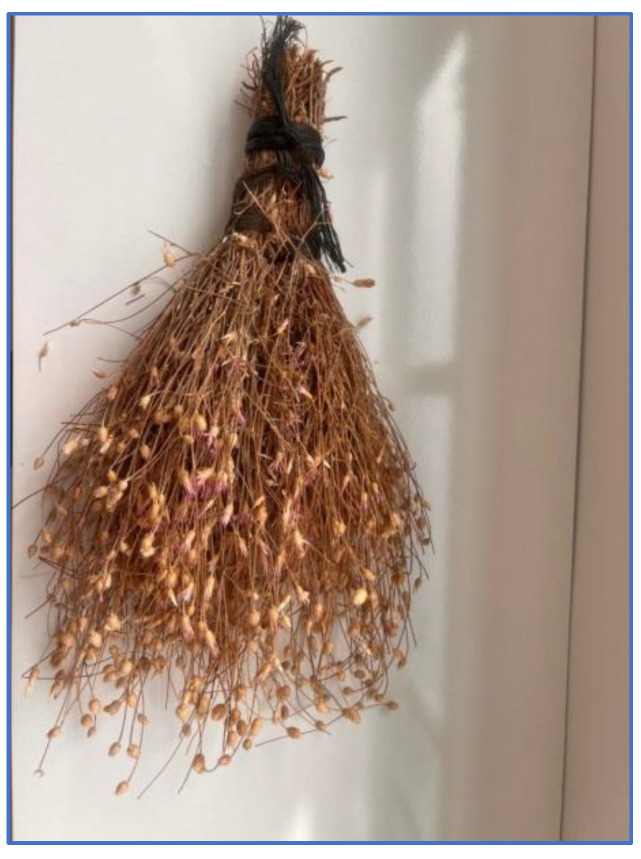
Broom made from *Xeranthemum annuum* (G. Emre, 2022).

The production of brooms from the aerial parts of the *Xeranthemum annuum* L. plant is noteworthy in the area (13 URs) (Figure 6).

**Figure 7:**
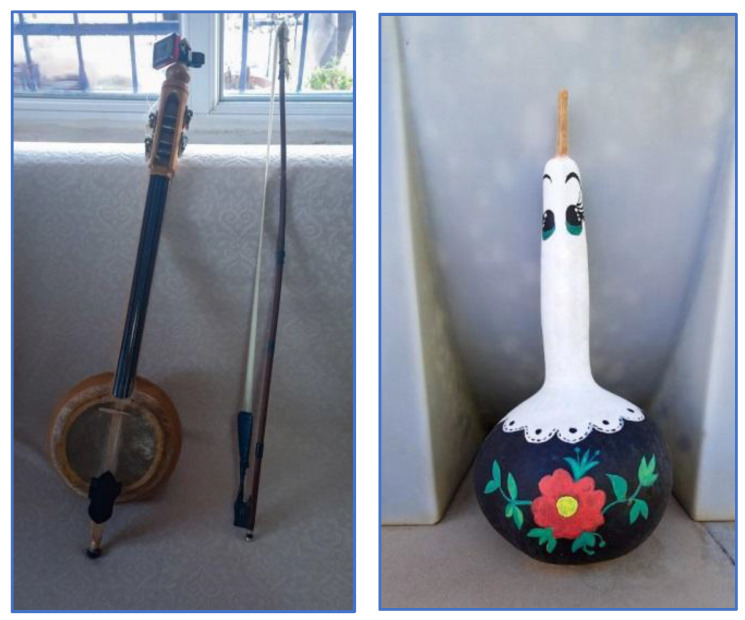
Musical instrument and ornament made from *Lagenaria siceraria* (İ. Yılmaz, 2022).

An interview with a family of musicians in Sarıuşağı Village revealed that they utilized some plants to make musical instruments. These plants are *J. regia*, *Lagenaria siceraria* (Mol.) Standl., and *Morus alba* L. The instrument crafted from *L. siceraria* is commonly referred to as a ‘kabak keman’ (gourd violin). *L. siceraria* is also utilized as an ornamental plant in the region (Figure 7).

### 2.4. Plant Names

The survey also inquired about the local names of therapeutic plants. The adoption of the same vernacular name for multiple plant species in a given location might lead to confusion. Documented instances of multiple namings are provided in Table 2. Since some of the people we interviewed remembered the Kurdish names of some plants, we recorded these names. These are: *Crocus chrysanthus* (Herbert) Herbert -Bivangk, Bivongk, *Descurainia sophia* (L.) Webb ex Prantl-Şıjıng, *E. angustifolia*- Dara bi, *E. macroclada*-Xaşule, *Gundelia tournefortii*-Gareng, *M. neglecta*-Toluk, *Mentha longifolia* (L.) Hudson subsp. *typhoides* (Briq.) Harley-*M.* x *piperita* L. Pung, Pungha and, *Rumex cristatus* DC.-Sımask. In the course of our research, we discovered plants that serve no purpose other than being referred to by their local name. These are the following *Delphinium ajacis* L. (MARE 22814)-Keloğlan, Mor menekşe, *Reseda lutea* L. (MARE 22809)-Eşek turpu, and *Phlomis armeniaca* Willd. (MARE 22740)-Susam otu.

### 2.5. Animal Usages

There is recorded information regarding the utilization of animal-derived products for several purposes throughout the region. Among these purposes, the quantity of therapeutic applications is particularly notable. Tuberculosis is the most common disease for these applications. As an instance, patients consumed cooked hedgehog and puppy meat, as well as donkey milk and turtle blood. Hedgehog meat is also utilized to treat hemorrhoids and rheumatism, and as an analgesic. Donkey blood is administered internally to treat eczema. Cooked turtle meat is consumed as food and is also utilized to treat warts. Rabbit fat applied to the ear is used to alleviate earaches. Prepared beef tongue is used to treat youngsters with delayed speech development. There are further data regarding the utilization of animal-derived resources for many other purposes in the area. Boiled turkey excrement is used in a poultice to alleviate irritated wounds. Buffalo horns are commonly used as containers to store oil. Catfish skin is used to make the body of the ‘kabak keman’ by cutting the *L. siceraria* fruit lengthwise, emptying it and drying it, and then stretching it. Horsehair is used in the production of traps and violin bows.

## 3. Materials and Methods

### 3.1. Study Area

**Figure 8:**
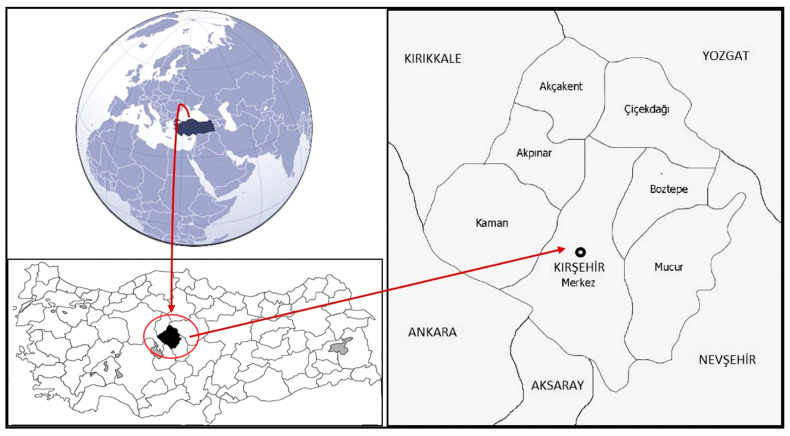
Map of Kırşehir.

Kırşehir is a province in Central Anatolia, located at a latitude of 39°19′ N and a longitude of 34°8′ E, covering an area of 6584 km^2^. Its elevation is 985 m a.s.l. It borders Nevşehir to the east and southeast, Aksaray to the south, Kırıkkale to the west, Ankara to the southeast, and Yozgat to the north and northeast. It has a total of seven districts and 252 villages. These districts are Akçakent, Akpınar, Boztepe, Çiçekdağı, Kaman, Center, and Mucur (Figure 8).

**Figure 9:**
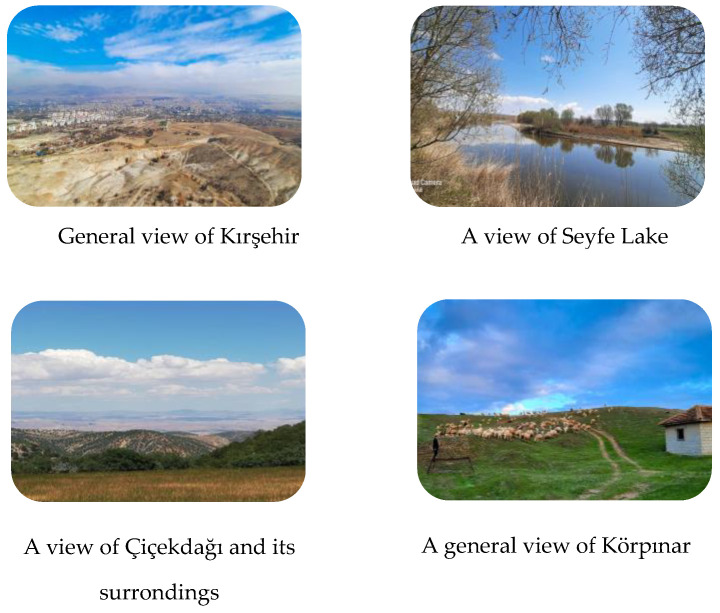
General views of Kırşehir and its surroundings (İ. Yılmaz, 2021).

The Kırşehir Massif, also referred to as a plateau, is surrounded by mountain ranges. Lake Seyfe is located on this plateau. The province is 65% plateaus, 17% mountains, and 18% plains (Figure 9).

The Kızılırmak, Delice, Kılıçözü, and Kaman rivers, as well as the Seyfe, Kesikköprü, and Çuğun reservoirs, are significant water sources in the region. The Central Anatolian region of Kırşehir experiences a typical continental climate. Winters exhibit low temperatures and frequent rainfall, while summers exhibit high temperatures and aridity. The mean annual precipitation is 383.3 mm [53].

Kırşehir has the effect of the Iran-Turan Flora region and is dominated by plant elements belonging to annual or perennial herbaceous steppe vegetation. Steppe areas have been formed due to the use of trees in some forests as fuel by humans. Most species of the Asteraceae, Fabaceae, Lamiaceae, and Poaceae families are found in steppe areas. The most common genera in steppe areas are the thorny *Astragalus* L. and *Eryngium* L. Kırşehir also has forest vegetation, to a lesser extent. Although *Quercus* L. trees are naturally distributed in forest vegetation areas, *Pinus* L. and *Cedrus* Trew trees have been grown as plantations. In addition, species belonging to the genus *Salix* L., *Populus* L. and *Tamarix* L. are distributed along the streams [54,55,56].

**Figure 10:**
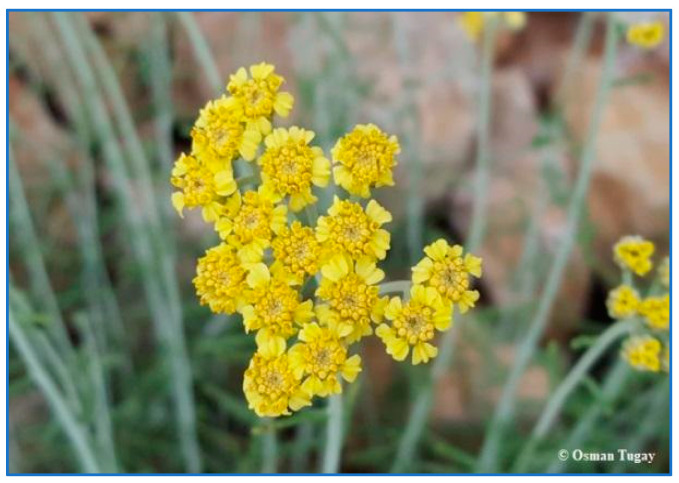
*Achillea lycaonica* (O. Tugay, 2021).

**Figure 11:**
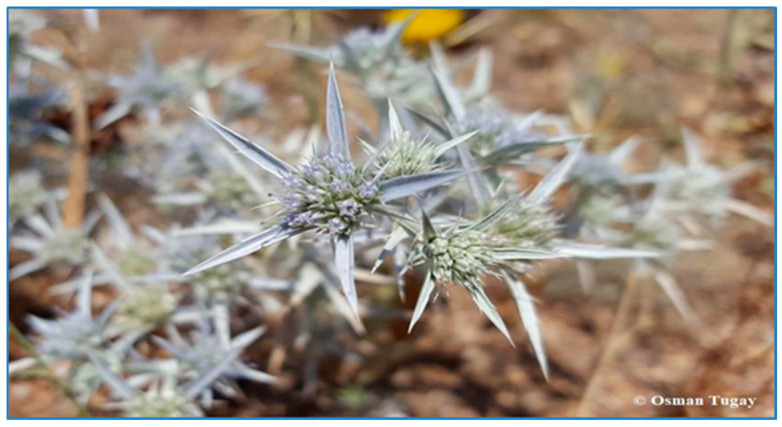
*Eryngium bithynicum* (O. Tugay, 2021).

Some plants in Kırşehir are endemic to Türkiye, e.g., *Achillea lycaonica* Boiss. et Heldr., *Astragalus densifolius* Lam., *Astragalus condensatus* Ledeb., *Centaurea kotschyi* (Boiss. et Heldr.) Hayek var. *kotschyi*, *Cousinia halysensis* Hub.-Mor., *Dianthus anatolicus* Boiss., *Eryngium bithynicum* Boiss, *Glaucium grandiflorum* Boiss. et Huet var. *torquatum* Cullen, *Onopordum turcicum* Danin, *Phlomis nissolii* L., and *Salvia virgata* Jacq. (Figure 10 and Figure 11).

### 3.2. Field Study

**Figure 12:**
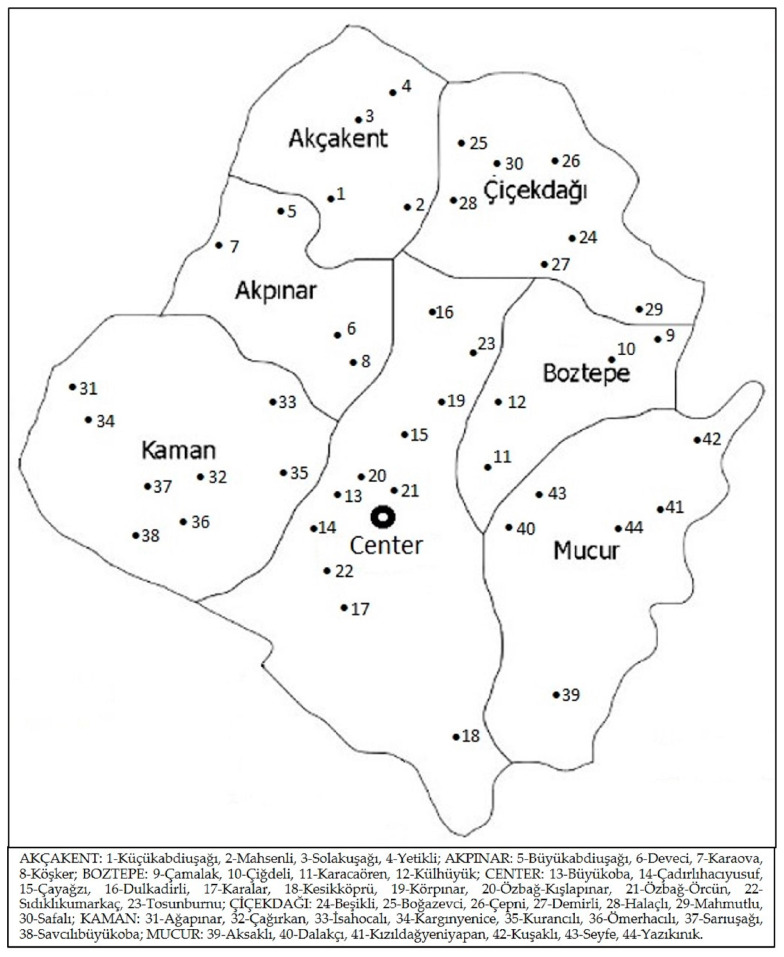
Map of villages visited.

The study was conducted in accordance with the ethical guidelines set forth by the American Anthropological Association Code of Ethics and the International Society of Ethnobiology Code of Ethics [57,58]. Data were mostly gathered utilizing the free listing technique, supplemented by strolls with chosen key informants, from June 2021 to June 2022, predominantly during the spring. The duration of our field trip was 44 days, during which we visited a total of 44 villages (Figure 12). We applied a snowball sampling approach, asking the informants to indicate further people experienced in traditional plant use. All interviews in all settlements were conducted in Turkish.

The informants had various occupations: farmers, housewives, shepherds, mukhtar (village headmen), and laborers (forestry workers). Interviews were conducted in coffee houses, gardens, houses, fields, etc. The sources of information and data included knowledgeable adults and patients. They provided details such as local names, specific parts of plants utilized, diseases treated, therapeutic effects, methods of preparation, methods of administration, and other plant usages (Appendix A). Throughout this research, with the assistance of our veterinarian colleague, our team also documented certain ancestral knowledge pertaining to animals.

When the villages or localities where fieldwork was conducted were evaluated from a socio-economic perspective, it was observed that agriculture and animal husbandry were important in terms of livelihood. It is possible to make the two following demographic observations about Kırşehir Province. As in Türkiye in general, the population in some villages is elderly. Although the majority of the population are literate, most were not educated beyond primary school. In order to access traditional knowledge, elderly individuals were favored as resource people.

The field study was designed to take into account the ethnic structure of Kırşehir. Of the communities examined in the field study, 28 are Turkmen or Yoruk settlements. Four settlements are predominantly Kurdish, and two of these are inhabited by the Abdal, who are of Çepni Turkish descent. Four settlements are Tatar. Diverse ethnic groups coexist in six settlements.

From a total of 99 people interviewed in the settlements during the field studies, 63 of these people (Table 3) were aged 50–70. It was observed that 25 people were aged 70 and over and constituted the second largest group. The ratio of women to men in this age group was approximately 1:2. As observed during the fieldwork, individuals over age 50 have more traditional knowledge than younger individuals. These people have knowledge of how their elders benefit from plants, even if they do not use them themselves. It is understood from field experience that interest in traditional knowledge has decreased, especially since the 2000s. Macro-level social changes, the rate of urbanization, the increase in transportation facilities, the proliferation of communication channels, and the socialization of medicine can be considered as factors contributing to the decreasing interest in traditional knowledge. However, some of the participants think that the knowledge they learned from past generations is very important now, especially due to recent increased interest in plants.

**Figure 13:**
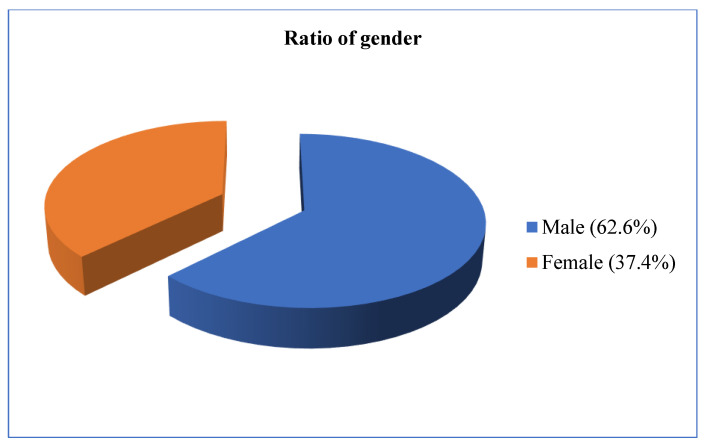
Gender features of informants.

When we look at the gender distribution of the field studies’ informants, we see that there are more male (62 males–62.6%) than female participants (37 females–37.4%) (Figure 13).

The collected plants were identified by the authors (G.E., I.S.), using *The Flora of Türkiye and the East Aegean Islands* [54,55,56] and *Illustrated Flora of Türkiye Vol 2* [59]. Voucher specimens were deposited at the Herbarium of the Faculty of Pharmacy, Marmara University (MARE). Taxonomic changes according to Plants of the World Online [60] are shown in parentheses with scientific names in Table 1.

As stated by Heinrich et al. [61,62], our results include only primary data. These data consist of the total number of use reports (URs), which represents the number of individual citations of a plant taxon. We established a database that encompasses the taxon (including family), local name, parts utilized, preparation method, administration technique, documented usage, and overall count of URs. In order to classify the ailments treated with plants mentioned in the interviews, we utilized a symptom-based nosological approach, which is a regularly used method in ethnobotanical research [2].

## 4. Conclusions

This study presents a thorough examination of ethnobotanical knowledge in Kırşehir, showcasing the wide range of ways in which plant species are used for medicinal, food, and other purposes. The traditional applications of these plants exemplify their deep-rooted cultural legacy and fundamental significance in the lives of the local cultures. The documenting of recent ethnobotanical discoveries enhances our comprehension of traditional plant utilization in this area, underscoring the significance of safeguarding and advocating for this invaluable knowledge for future generations. The information documented in these ethnobotanical studies increases our scientific knowledge and understanding, helps prepare a foundation for the sustained and widespread utilization of beneficial but heretofore overlooked plant species, and identifies plant species which may have the potential—great or small—to improve our nutrition and health care.

## Figures and Tables

**Table 1:** Ethnobotanical usages of plants in Kırşehir (Türkiye).

Botanical Name, Family and Voucher Specimen Number	Local Name	Plant Part Used (Medicine, Food or other Usages)	Ailments Treated/ Therapeutic Effect, Modes of Consumption or Usages	Preparation and Administration	Use Reports	References
*Achillea biebersteinii* Afan. [*Achillea arabica* Kotschy.] (Asteraceae, MARE 22745, 22854)	Sıçan otu	Aerial parts	Wound	Crushed, ext.	12	Med [10,12,15,17] ^b^
		Aerial parts	Ornament		5	
*Achillea wilhelmsii* C.Koch [*Achillea santolinoides* Lag. subsp. *wilhelmsii* (K.Koch) Greuter] (Asteraceae, MARE 2272, 22746)	Sıçan otu	Aerial parts	Wound	Crushed, ext.	14	Toothache [12] Med [20] ^b^
		Aerial parts	Toothache	Mouthwash, ext.	4	
*Allium cepa* L. ^a^ (Amaryllidaceae, MARE 15136)	Soğan	Bulbus	Wound	Digged into the ashes, ext.	8	Wound [14,24] Med [12,19,21] ^b^
		Bulbus	Sprain	Digged into the ashes, ext.	3	
		Bulbus	Fracture	Crushed, mixed with egg, and wrapped in a cloth, ext.	3	
		Bulbus	Gynecological diseases	Decoction, int.	1	
		Tunic	Abortive	Decoction, int.	1	
		Tunic	Dye		12	
*Amaranthus* sp. (Amaranthaceae, MARE 22767, 22782)	Karagöz	Aerial parts	Cooked		10	Food [20]
*Anchusa leptophylla* Roemer et Schultes subsp. *leptophylla* [*Anchusa leptophylla* Roem. et Schult.] (Boraginaceae, MARE 22797)	Sormuk otu	Nectar	Children snack		2	Food [9,16,22]
*Anchusa undulata* L. subsp. *hybrida* (Ten.) Coutinho (Boraginaceae, MARE 22804)	Sarmuk	Nectar	Children snack		5	Food [22,25]
*Astragalus melanophrurius* Boiss. ^e^ (Fabaceae, MARE 22824)	Geven, Keven	Latex	Hand-foot cracks	─, ext.	10	
		Latex	Wound	─, ext.	14	
		Latex	Fracture	─, ext.	3	
		Roots	Fuel	─, ext.	4	
		Aerial parts	Fodder		3	
*Beta lomatogona* Fisch. et Mey. (Amaranthaceae, MARE 22764)	Kızılca, Kızılca pancarı	Aerial parts	Boiled then cooked		4	Food [13]
*Brassica deflexa* Boiss. (Brassicaceae, MARE 22866)	Sarı hardal	Young leaves	Raw		9	
		Young leaves	Raw in salads		9	
*Capsella bursa-pastoris* (L.) Medik. (Brassicaceae, MARE 22844)		Aerial parts	Raw		8	Food [9,16,18,20,22,25]
*Capsicum annuum* L. ^a^ (Solanaceae, MARE 21420)		Fruits	Cough	Crushed, mixed with melted beef suet and molasses, int.	2	Med [14,21] ^b^
		Fruits	Cough	Crushed and wrapped around the chest, ext.	4	
*Cardaria draba* (L.) Desv. [*Lepidium draba* L.] (Brassicaceae, MARE 22739)	─	Aerial parts	Fodder		2	
*Carthamus tinctorius* L. (Asteraceae, MARE 22803)	Aspir	Oleum	Fuel		14	
*Centaurea depressa* Bieb. (Asteraceae, MARE 22786, 22787)	Düğmecik otu, Gökbaş	Capitula	Flower crown		16	
		Aerial parts	Fodder		1	
*Centaurea iberica* Trev. ex Sprengel (Asteraceae, MARE 22820)		Capitula	Wound	Crushed then boiled, ext.	4	[15,20] ^b^
*Centaurea solstitialis* L. subsp. *solstitialis* (Asteraceae, MARE 22736, 22813)	Boz diken, Çakırdiken, Sarıbaş	Capitula	Abdominal pain	Crushed, int.	5	Constipation [13] Med [23] ^b^
		Capitula	Constipation	Decoction, int.	3	
*Centaurea urvillei* DC. subsp. *stepposa* Wagenitz ^e^ (Asteraceae, MARE 22795)	Kadıgöbeği, Köygöçüren	Roots	Carminative	Decoction, int.	2	
		Receptaculum	Cooked		1	
*Centaurea virgata* Lam. (Asteraceae, MARE 22788)	Acımık otu	Capitula	Flower crown		9	
		Capitula	Used to remove louse from clothing		1	
*Chenopodium album* L. subsp. *album* var. *album* [*Chenopodium album* L.] (Amaranthaceae, MARE 22859)	Kızılca, Sirken	Aerial parts	Boiled then cooked		6	Food [9,10,16,18,20,22]
		Aerial parts	Boiled then made börek		6	
		Aerial parts	Soup		4	
*Chondrilla juncea* L. (Asteraceae, MARE 22868)	-	Latex	Chewed		5	Food [18,22,25]
*Cichorium intybus* L. (Asteraceae, MARE 22794)	Çıtlık	Latex	Wound	Ext.	1	Wound [10] Med [13] ^b^ Food [9,16,22,25] Broom [9]
		Young shoots	Boiled and served with eggs, fried with (or without) eggs		11	
		Young shoots	Raw		2	
		Latex	Chewed		4	
		Aerial parts	Broom		6	
*Citrullus lanatus* (Thunb.) Matsum. et Nakai. ^a^ (Cucurbitaceae, MARE 22248)	Karpuz	Pericarpium	Toy		2	
*Convolvulus galaticus* Rostan ex Choisy ^e^ (Convolvulaceae, MARE 22808)	Dağ sarmaşığı	Leaves	Wound	Crushed wrapped in a cloth, ext.	1	Fodder [25]
		Aerial parts	Fodder		7	
*Crataegus monogyna* Jacq. subsp. *azarella* (Gris.) Franco [*Crataegus monogyna* Jacq.] (Rosaceae, MARE 22845)	Alıç	Fruits	Shortness of breath	Decoction, int.	2	
		Fruits	Raw		8	
		Fruits	Colorant for desserts		1	
*Crocus chrysanthus* (Herbert) Herbert (Iridaceae, MARE 16649)	Bivangk (K), Bivongk (K), Çiğdem, Katırçiğdemi	Whole plant	Raw		3	Food [22]
		Corm	Raw		3	
		Flowers	Recreational tea		2	
		Flowers	Jam		2	
*Cydonia oblonga* Mill. (Rosaceae, MARE 23350)	Ayva	Leaves	Cold	Decoction, int.	16	Shortness of breath [13,14,24] Cold [19] Med [10,15,21] ^b^ Food [25]
		Leaves	Shortness of breath	Decoction, int.	6	
		Leaves	Diarrhea (in animals)	Decoction, int.	4	
		Leaves	Recreational tea		11	
		Fruits	Cooked (stuffed quince)		13	
*Descurainia sophia* (L.) Webb ex Prantl (Brassicaceae, MARE 22758)	Kaba süpürge, Karabacak, Şıjıng (K)	Aerial parts	Broom		2	
*Echium italicum* L. (Boraginaceae, MARE 22805)	Kurtkuyruğu, Tilkikuyruğu	Young shoots	Raw		4	Food [16,20,22]
*Elaeagnus angustifolia* L. ^a^ (Elaeagnaceae, MARE 22749, 22853)	Dara bi (K), İğde	Leaves	Boils	Wrapped in a cloth, ext.	5	Boil [14] Med [15,17,19,21] ^b^ Food [25]
		Leaves	Abdominal pain (in animals)	Wrapped in a cloth, ext.	1	
		Fruits	Raw		14	
		Young shoots	Amulet		18	
		Flowering branches	Fragrant		3	
*Eryngium campestre* L. var. *virens* Link (Apiaceae, MARE 22778, 22790, 22867)	Şeker dikeni, Yer kestanesi	Young stem	Peeled then eaten		6	Food [9,16,20]
		Roots	Rennet		1	
*Euphorbia macroclada* Boiss. (Euphorbiaceae, MARE 22811)	Sütleğen, Xaşule (K)	Latex	Wound	─, ext.	6	Wart [23] Callus [15] Med [20] ^b^
		Latex	Blood stopper	─, ext.	1	
		Latex	Malaria	Prepared a poultice with barley flour, ext.	1	
		Latex	Warts	─, ext.	1	
		Latex	Calluses	─, ext.	11	
		Latex	Tattooing		3	
*Glaucium grandiflorum* Boiss. et Huet. (Papaveraceae, MARE 22806)	Gelincik	Petals	Toy		1	
*Gundelia tournefortii* L. (Asteraceae, MARE 22865)	Kenger, Gareng (K)	Latex	Gum diseases	Chewed	1	Med [9,10] ^b^ Food [9,10,20,22,25]
		Shoots	Cooked		4	
		Capitula	Soup		2	
		Latex	Chewed		6	
		Fruits	As coffee		4	
		Receptaculum	Cooked		1	
		Young stem	Peeled then eaten		4	
*Hyoscyamus reticulatus* L. (Solanceae, MARE 22857)	─	Seeds	Against itching in the eyes	Seeds are spread on dying embers and eyes are exposed to fume	2	Against itching in the eyes [15] Med [23] ^b^
*Juglans regia* L. ^a^ (Juglandaceae, MARE 22748)	Ceviz	Leaves	Abdominal pain	Maseration with water, int.	1	Abdominal pain [17] Med [13,19,20,21,23,24] ^b^ Dye [20] Food [25]
		Leaves	Foot odor	Infusion, ext.	1	
		Leaves	Headache	Crushed and mixed with henna, ext.	2	
		Leaves	Vermifuge	Decoction, int.	1	
		Pericarp	Headache	Crushed, ext.	1	
		Pericarp	Hair remover	Ash is used to make a paste with water, ext.	1	
		Seed	Raw		12	
		Leaves	Dye		10	
		Pericarp	Dye		10	
		Endocarp	Toy		1	
		Wood	Musical instrument		1	
*Kochia scoparia* (L.) Schrad. [*Bassia scoparia* (L.) A.J. Scott] (Amaranthaceae, MARE 15200)	Çalgı	Aerial parts	Broom		18	
*Lactuca serriola* L. (Asteraceae, MARE 22747, 22862)	Çıtlık	Young leaves	Cooked		6	Food [16,20,22,25]
		Young leaves	Raw in salads		4	
		Young leaves	Choped and added into a mixture of yogurt + water to make ‘Cacık’		3	
		Latex	Chewed		2	
		Aerial parts	Fodder		2	
*Lagenaria siceraria* (Mol.) Standl. ^a^ (Cucurbitaceae, MARE 9639)	Süs kabağı	Fruits	Musical instrument		1	
		Fruits	Ornament		1	
*Lycium depressum* Stocks (Solanaceae, MARE 22773)	Çalı	Branches	Wart	─, ext.	9	
*Lycopersicon esculentum* Miller ^a^ (Solanaceae, MARE 23351)	Domates	Fruits	Athlete’s foot	Crushed wrapped in a cloth, ext.	2	Burn [21] Med [14] ^b^
		Fruits	Scorpion bite	Crushed and boiled (paste), ext.	6	
		Dried fruit	Snow burn	Boiled, ext.	3	
		Dried fruit	Burn	Boiled in water, ext.	1	
*Malva neglecta* Wallr. (Malvaceae, MARE 22753, 22829)	Çobançöreği, Sultanmercimeği, Toluk (K)	Aerial parts	Hemorrhoids	Decoction, ext.	7	Abdominal pain [20] Hemorrhoids [13,14,20,23] Wound [17] Gynocological disases [9] Med [12,15,21] ^b^ Food [9,16,18,20,22]
		Aerial parts	Expectorant	Infusion, int.	1	
		Aerial parts	Wound	Roasted and prepared a poultice with flour, ext.	14	
		Aerial parts	Abdominal pain	Boiled with bulgur and wrapped in a cloth, ext.	16	
		Leaves	Abdominal pain	Infusion, int.	2	
		Leaves	Abdominal pain	Roasted and wrapped in a cloth, ext.	8	
		Leaves	Gynecological diseases	Infusion, int.	1	
		Roots	Infertility	Boiled and sit against the steam	2	
		Roots	Menstrual disorders	Decoction, int.	1	
		Young leaves	Cooked		20	
		Young leaves	Raw		2	
		Immature fruits	Children snack		4	
*Malva sylvestris* L. (Malvaceae, MARE 22753a)	Ebegümeci, Evelik	Roots	Gynecological diseases	Ext.	1	Med [10] ^b^ Food [18,22]
		Leaves	Wound	Cooked with flour, wrapped in a cloth, ext.	17	
		Leaves	Abdominal pain (in babies)	Cooked then wrapped in a cloth, ext.	4	
		Leaves	Gynecological diseases	Cooked, ext.	1	
		Young leaves	Cooked		20	
		Leaves	Dolma		8	
		Immature fruits	Children snack		4	
*Matricaria chamomilla* L. var. *recutita* (L.) Grierson (Asteraceae, MARE 22850, 22873)	Papatya	Capitula	Sore throat	Inhalation	2	Med [14,24] ^b^ Recreational tea [20]
		Capitula	Gynecological diseases	Infusion, int.	2	
		Capitula	Recreational tea		9	
*Mentha longifolia* (L.) Hudson subsp. *typhoides* (Briq.) Harley (Lamiaceae, MARE 22860)	Pung (K), Pungha (K), Yarpız	Aerial parts	Headache	Wrapped in a cloth, ext	3	Med [12,13,14,21,23] ^b^ Food [9,16,18,20,22]
		Leaves	Spice		17	
		Aerial parts	Dye		4	
		Leaves	Used as a soap (foamed with water after crushed)		2	
*Mentha* x *piperita* L. ^a^ (Lamiaceae, MARE 22863)	Pung (K), Pungha (K), Nane	Leaves	Spice		21	Food [18]
*Morus alba* L. ^a^ (Moraceae, MARE 23348)	Dut	Leaves	Eczema	Boiled, wrapped in a cloth, ext	1	
		Leaves	Dolma		1	
		Branches	Walking stick		2	
		Branches	Musical instrument		1	
*Nasturtium officinale* R.Br. (Brassicaceae, MARE 22852)	Kusuk	Leaves	Raw in salads		7	Food [10,20]
*Papaver argemone* L. [*Roemeria argemone* (L.) C. Morales, R. Mend. et Romero García] (Papaveraceae, MARE 22830)	Gelincik	Petals	Jam		4	Food [16]
		Aerial parts	Cooked		4	
*Papaver pilosum* Sibth. et Sm. ^e^ (Papapveraceae, MARE 22840)	Gelincik	Petals	Jam		4	Food [16]
*Papaver rhoeas* L. (Papaveraceae, MARE 22756a)	Gelincik	Petals	Jam		4	Food [16,22]
		Aerial parts	Cooked		4	
*Peganum harmala* L. (Nitrariaceae, MARE 22743, 22779)	Üzerlik	Aerial parts	Eye infections	Inhalation	1	Med [10,12,15,17,19] ^b^ Amulet [9,10]
		Seeds	Amulet		10	
		Fruits	Amulet (strung on a rope)		28	
		Aerial parts	Amulet (hung on the wall in bunches)		28	
		Whole plant	Ashes were used to wash clothes		1	
		Fruits	Toy		1	
		Aerial parts	Incense		11	
		Fruits	Incense		9	
*Plantago lagopus* L. (Plantaginaceae, MARE 22733)	Kırksinir	Leaves	Wound	Wrapped in a cloth, ext.	9	
*Plantago major* L. subsp. *intermedia* (Gilib.) Lange (Plantaginaceae, MARE 22737, 22856)	Kırksinir, Yedidama rotu	Leaves	Wound	Wrapped in a cloth, ext.	9	Wound [14,15,21] Med [12,13,20,23] ^b^
		Leaves	Bronchitis (in children)	Mixed with honey, int	1	
		Leaves	Hemorrhoids	Boiled, int.	3	
		Leaves	Abdominal pain	─, int.	1	
		Leaves	Stomachache	─, int.	1	
		Leaves	Headach	─, int.	1	
*Polygonum cognatum* Meissn. (Polygonaceae, MARE 22810, 22861)	Madımak, Madımalak	Aerial parts	Cooked		26	Food [9,10,13,16,18,20,22]
		Aerial parts	Stuffing in pastries		4	
		Aerial parts	Raw in salad		8	
		Aerial parts	Dye		1	
*Populus* sp. (Salicaceae, MARE 23345)	Kavak	Bark	Fracture	Crushed, added egg then wrapped in a cloth, ext.	1	Med [15] ^b^
		Branches	Stick		2	
		Branches	Whistle		2	
		Wood	Fuel		1	
*Portulaca oleracea* L. (Portulacaceae, MARE 22771, 22781)	Semiz otu, Soğukluk otu	Aerial parts	Cooked		11	Food [14,16,18,20,22]
		Aerial parts	Raw in salads		11	
*Pyrus elaeagnifolia* Pallas subsp. *elaeagnifolia* Pallas [*Pyrus elaeagnifolia* Pall.] (Rosaceae, MARE 22741)	Ahlat, Çördük	Fruits	Diarrhea	─, int.	3	Diarrhea [21] Med [24] ^b^ Food [9]
		Fruits	Pickle		5	
		Fruits	Raw		1	
		Branches	Stick		1	
*Pyrus elaeagnifolia* Pallas subsp. *kotschyana* (Boiss.) Browicz (Rosaceae, MARE 22846)	Ahlat, Çördük	Fruits	Diarrhea	─, int.	3	
		Fruits	Pickle		5	
		Fruits	Raw		1	
*Quercus* sp. (Fagaceae, MARE 15063)	Meşe, Pelit	Fruit	As coffee after roasted and ground		1	
		Fruit	Roasted		6	
		Fruit	Ornament		2	
		Ashes	Washing cloths		4	
*Rosa canina* L. (Rosaceae, MARE 22754)	İtburnu, Kuşburnu	Fruits	Cold	Infusion, int.	16	Cold [13,14,17,19,23,24] Diabetes [12,15] Med [20,21] ^b^ Food [9,18,22]
		Fruits	Cough	Infusion, int.	9	
		Fruits	Immunostimulant	Infusion, int.	2	
		Fruits	Diabetes	Infusion, int.	1	
		Fruits	Recreational tea		7	
		Fruits	Jam		4	
*Rubus* sp. (Rosaceae, MARE 23344)	Böğürtlen	Branches	Boil	Burned after mixed with water, ext.	2	
		Fruits	Jam		12	
*Rumex cristatus* DC. (Polygonaceae, MARE 22796)	Ekşimen, Kuzukulağı, Sımask (K)	Leaves	Constipation	Cooked, int.	2	
		Leaves	Kidney stones	Decoction, int.	1	
		Leaves	Raw in salads		3	
		Leaves	Dolma		6	
*Rumex tuberosus* L. (Polygonaceae, MARE 22791)	Ekşimen, Kuzukulağı	Leaves	Raw in salads		2	Food [16]
		Leaves	Dolma		6	
*Salix alba* L. (Salicaceae, MARE 22793)	Söğüt	Leaves	Headache	Chewed	1	Med [13,14] Med [15] ^b^ Walking stick [9]
		Leaves	Antipyretic	Infusion, int.	3	
		Leaves	Antipyretic (in babies)	Decoction, int.	1	
		Flowers	Children snack		1	
		Branches	Basket		6	
		Branches	Walking stick		2	
		Branches	Whistle		2	
		Branches	Cigarette holder		1	
		Wood	Laundry stick		1	
		Wood	Toy		1	
		Wood	Fuel		1	
*Scrophularia* sp. (Scrophulariaceae, MARE 22752)	Girişik otu, Kaşıntı otu	Aerial parts	Antifungal	Decoction, ext.	1	
*Silene vulgaris* (Moench) Garcke (Caryophyllaceae, MARE 22792)	─	Fruits	Toy		3	
		Aerial parts	Fodder		1	
*Solanum tuberosum* L. ^a^ (Solanaceae, MARE 23346)	Gumbıl, Kumpir, Patates	Tubers	Headache	Sliced, wrapped in a cloth, ext.	2	Headache [12,21]
		Tubers	Mumps	Wrapped in a cloth after boiled and mashed, ext.	2	
*Teucrium polium* L. (Lamiaceae, MARE 22774, 22776, 22802, 22855)	Muradi, Periyavşan otu, Yavşan otu	Aerial parts	Abdominal pain	Bath, ext.	16	Appetizer [9,12,15,20] Med [10,14,17,21] ^b^
		Aerial parts	Itching	Bath, ext.	11	
		Aerial parts	Appetizer	Decoction, int.	2	
*Thymus longicaulis* C. Presl subsp. *longicaulis* C. Presl var. *subisophyllus* (Borbas) Jalas [*Thymus longicaulis* subsp. *chaubardii* (Boiss. et Heldr. ex Rchb.f.) Jalas] (Lamiaceae, MARE 22760)	Kekik	Aerial parts	Sorethroat	Infusion, int.	4	Food [13] Med [14]
		Aerial parts	Shortness of breath	Infusion, int.	6	
		Aerial parts	Cough	Infusion, int.	5	
		Aerial parts	Spice		11	
		Aerial parts	Recreational tea		7	
*Thymus sipyleus* Boiss. subsp. *rosulans* (Borbás) Jalas [*Thymus sipyleus* Boiss.] (Lamiaceae, MARE 22858)	Kekik	Aerial parts	Cold	Decoction, int.	8	Cold [19] Med [12,15] ^b^ Food [22]
		Aerial parts	Spice		10	
		Aerial parts	Recreational tea		5	
*Tragopogon dubius* Scop. (Asteraceae, MARE 22789, 22801)	Dedesakalı, Emlik, Tatazkalı, Tekecen	Young stem	Raw		8	
		Young stem	Soup		1	
*Tribulus terrestris* L. (Zygophyllaceae, MARE 22818)	Çobandiken	Aerial parts	Diabetes	Decoction, int.	1	Diabetes [10] Med [19,23] ^b^
		Aerial parts	Weight loss	Infusion, int.	1	
		Fruits	Weight loss	Infusion, int.	1	
*Tripleurospermum parviflorum* (Willd.) Pobed. (Asteraceae, MARE 22842)	Papatya	Capitula	Sore throat	Infusion, int.	4	Med [15,20] ^b^
		Capitula	Sore throat	Inhalation	3	
		Capitula	Gynecological disorders	Infusion, int.	2	
		Capitula	Recreational tea		3	
		Capitula	Flower crown		6	
*Urtica urens* L. (Urticaceae, MARE 22751)	Isırgan	Aerial parts	Rheumatism	Crushed, wrapped in a cloth, ext.	3	Shortness of breath [14] Rheumatism [15,23] Med [20] ^b^ Food [16,18,22]
		Aerial parts	Wound	Crushed, wrapped in a cloth, ext.	2	
		Aerial parts	Shortness of breath	Decoction, int.	1	
		Aerial parts	Constipation	Cooked, int.	1	
		Leaves	Hemorrhoids	─, ext.	1	
		Leaves	Cooked		5	
		Leaves	Soup		2	
		Leaves	Borek		4	
*Verbascum* sp. (Scrophulariaceae, MARE 22744)	Sığırkuyruğu, Zarmas	Aerial parts	Hemorrhoids	Decoction, int.	1	
		Flowers	Shortness of breath	Infusion, int.	3	
		Flowers	Wart	Crushed, ext.	1	
		Leaves	Hemorrhoids	Crushed, ext.	1	
		Aerial parts	Broom		6	
		Aerial parts	Fuel		2	
*Vicia cracca* L. (Fabaceae, MARE 22738, 22759)	Dağ yoncası, Fiğ	Fruits	Children snack		2	Food [16]
*Vitis vinifera* L. ^a^ (Vitaceae, MARE 15033)	Asma, Üzüm	Fruits (dried)	Fracture	Crushed, wrapped in a cloth, ext.	1	Fracture [10,14,15,24] [21] ^b^
		Branches	Eye diseases	Crushed and dropped into the eye	1	
		Leaves	Dolma		18	
		Fruits	Pekmez		18	
		Fruits	Dessert		20	
		Branches	Used as a lighter		3	
		Leaves	Dye (mixed with henna)		1	
*Xeranthemum annuum* L. (Asteraceae, MARE 22869)	Süpürge otu	Aerial parts	Broom		13	
*Zea mays* L. subsp. *mays* ^a^ (Poaceae, MARE 21466)	Mısır	Stylus	Antiinflammatory	Infusion, int.	1	Diuretic [14,15] Med [19,24] ^b^
		Stylus	Diuretic	Infusion, int.	2	

Abbreviations: Int.—Internal use; Ext.—External use; Med.—Medicinal usage. ^a^ Cultivated plant; ^b^ Different usages; ^e^ Endemic plant. The language of local names are in Turkish and Kurdish (K).

**Table 2:** List of same vernacular plant names used for multiple taxa.

Local Name	Botanical Names, Family and Specimen Numbers			
Sıçan otu	*Achillea arabica*	*Achillea wilhelmsii*		
Papatya	*Matricaria chamomilla* var. *recutita*	*Tripleurospermum parviflorum*		
Pung	*Mentha longifolia* subsp. *typhoides* var. *typhoides*	*Mentha* x *piperita*		
Gelincik	*Glaucium grandiflorum*	*Papaver argemone*	*Papaver pilosum*	*Papaver rhoeas*
Kırksinir	*Plantago lagopus*	*Plantago major* subsp. *intermedia*		
Ahlat, Çördük	*Pyrus elaeagnifolia* subsp. *elaeagnifolia*	*Pyrus elaeagnifolia* subsp. *kotschyana*		
Ekşimen, Kuzukulağı	*Rumex cristatus*	*Rumex tuberosus*		
Kekik	*Thymus longicaulis* subsp. *longicaulis*	*Thymus sipyleus*		

**Table 3:** Educational status of participants [W = Women, M = Men].

Age Range						
Education Status	<19–49		50–70		>70	
	W	M	W	M	W	M
Illiterate	0	0	0	0	6	2
Literate	1	0	3	6	4	4
Primary school graduate	1	1	20	19	0	7
Secondary School Graduate	1	1	0	9	0	2
High school graduate	1	2	0	4	0	0
Graduated from a University	1	2	0	2	0	0
	11		63		25	
Total	99					

W = Women, M = Men.

## Appendix A

Questionnaire Form

1. Name and surname of participant.
2. Age and gender of participant.
3. Telephone and address of participant.
4. Educational level of participant.
5. Date of interview.
6. Participant’s place of residence.
7. Duration of the participant’s stay at the current residance.
8. Local name of plant.
9. Human health or Animal health.
10. Ailments treated/therapeutic effect.
11. Plant part used.
12. Preparation.
13. Administration.
14. Dosage.
15. Duration of treatment.
16. Age group of patients (baby, child, adult).
17. Side effects.
18. Different ethnobotanical use.
